# Use of phosphodiesterase inhibitors and prevalence of self-reported glaucoma in the United States

**DOI:** 10.1371/journal.pone.0183388

**Published:** 2017-08-17

**Authors:** Stephanie P. Chen, Kuldev Singh, Shan C. Lin

**Affiliations:** 1 Department of Ophthalmology, Stanford University School of Medicine, Stanford, California, United States of America; 2 Department of Ophthalmology, University of California San Francisco, San Francisco, California, United States of America; Bascom Palmer Eye Institute, UNITED STATES

## Abstract

**Objective:**

While decreased ocular blood flow is thought to be a possible contributor to glaucoma pathogenesis, it is unclear what role systemic phosphodiesterase inhibitors (PDEi) play. We performed a cross-sectional study of a nationally representative sample of the U.S. population to investigate the relationship between the most commonly used PDEi, sildenafil and theophylline, and self-reported glaucoma.

**Methods:**

We used the National Health and Nutrition Examination Survey 2005–2008 cycles for this observational study. 7,042 participants, aged 40 years and over, responded to a survey item on glaucoma status and were included in the analysis. Multivariable logistic regression models were constructed to evaluate the association between at least 1 year of self-reported PDEi use and prevalent glaucoma. Regressions were adjusted for potential confounding variables, including demographics, socioeconomic status, and general health conditions, and accounted for the complex design of the survey. Sample weights were constructed and used to ensure the generalizability of results.

**Results:**

482 respondents self-reported a diagnosis of glaucoma, of which 11 used sildenafil and 20 used theophylline for at least 1 year. Covariates significantly associated with higher odds of glaucoma prevalence in univariable analyses included older age, black race, former smoking status, diabetes, hyperlipidemia, myocardial infarction, and stroke. Conversely, higher education and income were significantly associated with lower odds of glaucoma prevalence. In regression analyses adjusted for demographic and socioeconomic variables, sildenafil (OR = 4.90, CI: 1.24–19.27, p = 0.025) and theophylline (OR = 3.15, CI: 1.46–6.80, p = 0.005) were significantly associated with higher odds of self-reported glaucoma. These associations held after further adjustment with general health behaviors and conditions for both sildenafil and theophylline.

**Conclusions:**

Use of sildenafil and theophylline for one or more years was associated with greater prevalence of self-reported glaucoma, a finding which requires further prospective study to assess causality and possible mechanisms of action.

## Introduction

Glaucoma is a degenerative optic neuropathy and remains one of the leading causes of blindness in the United States and worldwide. At the beginning of the decade, prevalence of glaucoma in the United States was over 2 million, with numbers projected to increase to over 3 million by 2020 largely due to the aging population [1]. Estimates of the global prevalence of glaucoma were placed at over 57 million individuals in 2015, with 2020 estimates forecasted to rise over 65 million and 2040 estimates over 111 million [2,3]. Though primary open angle glaucoma (POAG) is the most common type of glaucoma, the exact mechanism of disease is still unclear. Damage to the retinal ganglion cell axons that comprise the optic nerve head is caused by a multitude of factors, most important among them elevated intraocular pressure (IOP), also the only known modifiable risk factor in glaucoma [4]. Hence, pharmaceutical and surgical therapies for glaucoma have exclusively targeted control of IOP, with variable success.

More recently, studies have demonstrated an association between altered, compromised ocular circulation and glaucoma, including a reduction in blood flow and subsequent oxidative stress around the optic nerve head in both POAG and normal tension glaucoma [5–8]. Systemic vasodilators, including some phosphodiesterase inhibitors (PDEi) have wide-ranging therapeutic purposes but may occasionally have unintentional consequences on vision. For instance, phosphodiesterase type 5 inhibitors (PDE5i), well known for applications in erectile dysfunction, can evoke transient visual changes in color perception and light sensitivity due to interference in retinal ganglion cell signaling [9,10].

Sildenafil is a PDE5i that is approved for use in the treatment of erectile disorder. Very rarely, there have been case reports of vision-threatening events after sildenafil use. These include non-arteritic ischemic optic neuropathy, angle-closure glaucoma, and optic atrophy [11,12]. Yet, because of sildenafil’s vasodilating actions, it is also conceivable that it can increase optic nerve blood flow and prevent or delay the development of glaucoma. Results have been mixed regarding the effects of sildenafil on ocular hemodynamics, with some studies showing increases in retrobulbar and choroidal blood flow while others have not [13–15]. Furthermore, it appears that acute sildenafil use has no effect on IOP and evidence is lacking for a role in the development and progression of glaucoma [14,16,17].

Theophylline, historically used as a bronchodilator to treat asthma and COPD, is not known to be associated with ocular side effects or any impact on glaucoma pathogenesis. Nevertheless, it is a non-selective PDEi and a xanthine derivative chemically similar to caffeine, which has been postulated to be associated with increased IOP in glaucoma patients [18–20].

In this study, we sought to investigate the association between PDEi use and prevalent glaucoma in a larger population sample using self-reported data from the National Health and Nutrition Examination Survey (NHANES). NHANES is an annual, nation-wide survey conducted by the National Center for Health Statistics (NCHS) as part of the Center for Disease Control and Prevention. Data is collected from the civilian, non-institutionalized population in the U.S. on health and nutrition. While glaucoma status defined by self-report has been viewed with some skepticism in the literature, we found the wealth of data available in NHANES uniquely suited to explore our hypothesis that use of PDEi medications is associated with lower odds of prevalent glaucoma in the United States.

## Methods

### Study sample

We performed a population-based study of a representative sample of American adults surveyed in the NHANES 2005–2008 cycles. Participants are selected through a complex procedure involving multistage probability sampling, with oversampling of certain subgroups. In the 2005–2006 cycle, over-samples include low-income persons, adolescents 12–19 years, person 60+ years of age, African Americans, and Mexican Americans [21]. In 2007–2008, NHANES oversampled the Hispanic population, and participants 40+ years of age saw an increase in numbers whereas 12–19 year-olds saw a decrease [22]. Sample weights are available to provide adjusted, unbiased data generalizable to the entire U.S. population.

### Measures

Glaucoma status was the primary outcome measure in the analysis. 7,081 participants in the 2005–2008 cycles were aged 40 years and over and eligible for additional questions on eye diseases in the vision portion of the NHANES interview. Participants were asked if an eye doctor has ever told them they have “glaucoma, sometimes called high pressure in your eyes”. Seven thousand and forty two reported whether they had been diagnosed with glaucoma (0.55% missing), and of these, 482 respondents reported a positive history of glaucoma.

Use of the major PDEi, sildenafil and theophylline, was the primary predictor variable of interest. Other PDEi were included in an initial screen, however too few participants in our study sample reported use of other PDEi for meaningful analysis, therefore we did not include them in the rest of our analysis. To ascertain medication use, participants were asked, “Have you taken or used any prescription medicines in the past month?” Though data on dosage was not collected, for those medications listed, participants were further asked, “For how long have you been taking this medicine?” Duration of use was converted to number of days and categorized to <1 year of use or ≥1 year of use. For analytic purposes, we included only participants who reported use of sildenafil or theophylline for ≥1 year.

Possible confounders included as covariates in our analyses included demographic and socioeconomic (SES) variables (self-reported age, gender, race-ethnicity, education level, and annual household income), and general health conditions and behaviors (self-reported cigarette smoking status–never, former, and current). Diabetes, hyperlipidemia (HLD), myocardial infarction (MI), and stroke status were based on self-reports of whether participants were told by a doctor or other health professional they have or had diabetes, high blood cholesterol, a heart attack, or stroke, respectively. Hypertension (HTN) status was ascertained from self-reports of whether a doctor told participants they have high blood pressure on 2 or more occasions.

For internal validation of self-reported glaucoma, we examined vertical cup-to-disc ratio (CDR) from the retinal imaging segment of NHANES and visual field loss from frequency doubling technology (FDT). We classified vertical CDR >0.7 in at least one eye and FDT defect in either eye as positive for glaucoma. FDT defect was defined as visual field abnormalities using a 2-2-1 algorithm incorporating test reliability indices per NHANES protocol [23].

### Statistical analysis

STATA/SE 13.1 (StataCorp, College Station, TX) was used to perform all analyses. We compared subgroups with and without glaucoma using survey-adjusted Rao–Scott–Pearson χ2 and Wald tests for categorical and continuous variables, respectively. A 4-year sample weight was constructed by combining the 2-year interview sample weights provided by NCHS for NHANES 2005–2008. This generates estimates representative of the population at the midpoint of the surveyed period. Variance estimates were derived using Taylor Series Linearization, as recommended by NCHS. Given the study design of NHANES, we used the svy set of commands in STATA.

Multivariable logistic regression models were used to determine the odds of prevalent glaucoma in respondents aged 40 years and over that used the PDEi sildenafil or theophylline for 1 year or more. All models were adjusted sequentially for demographics and SES (age, gender, race/ethnicity, education level, and annual household income) as well as general health conditions and behaviors (smoking status, diabetes, HTN, HLD, MI, and stroke). “Don’t know” and “Refuse” responses were considered missing values and excluded from the regression analyses. P-values <0.05 were deemed statistically significant.

## Results

Table 1 shows demographic, SES, and general health behaviors and conditions for the subgroups with and without glaucoma. Out of 7,042 participants aged 40 years and older who were asked about glaucoma, 482 (6.84%) reported a positive diagnosis of glaucoma. Compared to those without, those with self-reported glaucoma were significantly more likely to be older and have different racial-ethnic distributions, less education, and lower income. As well, they were significantly more likely to be former smokers and report histories of diabetes, hyperlipidemia, myocardial infarction, and stroke. These differences are corroborated with univariable analysis of covariates as illustrated in Table 2.

**Table 1:** Demographic and general health characteristics of participants age ≥40 years based on self-reported glaucoma status, in the National Health and Nutrition Examination Survey (NHANES) 2005–2008 (n = 7042).

Characteristic	Self-Reported Glaucoma		
	No (n = 6,560)	Yes (n = 482)	*P* Value*
Age, mean (SD), yr	56.54 (11.91)	66.86 (13.89)	<0.001
Gender (%)			
Female	52.95	52.38	0.868
Race/Ethnicity (%)			
Non-Hispanic White	75.88	72.46	0.001
Mexican/Hispanic	8.79	6.73	
Black	10.14	16.67	
Other	5.18	4.15	
Education (%)			
<High school graduate	19.00	27.51	<0.001
High school graduate/some college	53.86	57.03	
College graduate and beyond	27.14	15.46	
Annual household income (%)			
<$35,000	32.81	47.19	<0.001
≥$35,000 to <$65,000	25.74	31.24	
≥$65,000	41.45	21.57	
Smoking status (%)			
Never	49.43	45.71	<0.001
Former	29.85	41.97	
Current	20.72	12.32	
Health conditions (%)			
Diabetes	11.08	23.00	<0.001
Hypertension	84.83	86.18	0.617
Hyperlipidemia	47.81	58.85	<0.001
Myocardial infarction	5.02	9.80	0.005
Stroke	4.34	10.21	0.011
Tests/Imaging (%)			
FDT defect, yes	6.86	36.07	<0.001
FDT defect, no	93.14	63.93	
Vertical CDR >0.7	11.88	59.44	<0.001
Vertical CDR ≤0.7	88.12	40.56	

*FDT*: *frequency doubling technology*, *CDR*: *cup-to-disc ratio*. FDT defect defined as abnormal FDT findings in at least 1 eye.

*P values determined using the Wald test for age as a continuous variable and the Rao–Scott–Pearson χ2 test for all other categorical variables.

**Table 2:** Univariable analysis of self-reported glaucoma and possible risk factors in the National Health and Nutrition Examination Survey (NHANES) 2005–2008.

Characteristic	OR	95% CI	*P* value
Age	1.07	(1.05–1.08)	<0.001
Female sex	0.98	(0.74–1.29)	0.868
Race/Ethnicity			
White	—	—	—
Mexican/Hispanic	0.80	(0.59–1.09)	0.149
Black	1.72	(1.34–2.21)	<0.001
Other	0.84	(0.42–1.67)	0.606
Education			
<High school graduate	—	—	—
High school graduate or some college	0.84	(0.66–1.07)	0.156
College graduate and beyond	0.36	(0.25–0.52)	<0.001
Annual household income			
<$35,000	—	—	—
≥$35,000 to <$65,000	0.73	(0.56–0.96)	0.023
≥$65,000	0.39	(0.29–0.53)	<0.001
Smoking status			
Never	—	—	—
Former	1.52	(1.22–1.89)	<0.001
Current	0.64	(0.41–1.02)	0.059
Health conditions (%)			
Diabetes	2.40	(1.97–2.91)	<0.001
Hypertension	1.11	(0.71–1.76)	0.629
Hyperlipidemia	1.56	(1.31–1.86)	<0.001
Myocardial infarction	2.06	(1.42–2.98)	<0.001
Stroke	2.50	(1.43–4.39)	0.002
Tests/Imaging (%)			
FDT defect, yes	7.66	(5.41–10.86)	<0.001
Vertical CDR >0.7	10.87	(6.08–19.44)	<0.001

*FDT*: *frequency doubling technology*, *CDR*: *cup-to-disc ratio*. FDT defect defined as abnormal FDT findings in at least 1 eye. ORs are reported with 95% confidence intervals (CI) with two-sided *p-*values <0.05 deemed statistically significant.

In the internal validation analysis of self-reported glaucoma, 36% of respondents with self-reported history of glaucoma had visual field loss in either eye based on FDT abnormalities, and 60% had vertical CDR >0.7 in at least one eye (Table 1). On the other hand, only 7% of participants without self-reported history of glaucoma demonstrated FDT abnormalities and 12% had increased CDR. For both measures, the proportion was significantly higher in those who self-reported a history of glaucoma versus those who did not. Univariable analyses with FDT defect and CDR likewise showed strong, statistically significant associations with self-reported glaucoma (Table 2).

Table 3 shows results from the regression models evaluating the association between sildenafil and theophylline use and prevalent glaucoma. After ≥1 year of use, theophylline (n = 20) was significantly associated with higher odds of glaucoma (OR = 4.83, CI: 1.94–12.04, p = 0.001) whereas sildenafil (n = 11) was not (OR = 3.72, CI: 0.69–20.07, p = 0.122). When adjusted for demographic and SES variables (age, sex, race-ethnicity, education, and income), both sildenafil (OR = 4.90, CI: 1.24–19.27, p = 0.025) and theophylline (OR = 3.15, CI: 1.46–6.80, p = 0.005) use were significantly associated with higher odds of prevalent glaucoma. These associations persisted after further adjustment for health behaviors and conditions (smoking status, diabetes, hypertension, hyperlipidemia, myocardial infarction, and stroke) for the two medications.

**Table 3:** Logistic regression models for the association between self-reported glaucoma and use of sildenafil and theophylline, in the National Health and Nutrition Examination Survey (NHANES) 2005–2008.

Characteristic	OR	95% CI	*P* value
Sildenafil use (≥1 year of use)			
Unadjusted	3.76	(0.72–19.51)	0.111
Adjusted*			
Demographics and SES	5.07	(1.28–20.15)	0.023
Health behaviors/conditions	9.68	(2.13–44.02)	0.005
Theophylline use (≥1 year of use)			
Unadjusted	2.39	(0.42–13.55)	0.315
Adjusted*			
Demographics and SES	1.77	(0.34–9.31)	0.490
Health behaviors/conditions	3.87	(1.22–12.24)	0.023

*SES*: *socioeconomic status*. ORs are reported with 95% confidence intervals (CI) with two-sided *p-*values <0.05 deemed statistically significant.

*Adjusted models include sequential adjustments for demographics and SES (age, sex, race-ethnicity, education, income) and demographics + general health behaviors/conditions (smoking status, diabetes, hypertension, hyperlipidemia, myocardial infarction, stroke).

## Discussion

This investigation suggests that there may be an association between use of the PDEi sildenafil and theophylline (for at least 1 year) and glaucoma diagnosis. Though the mechanisms of association between glaucoma and sildenafil or theophylline use are likely quite different, to our knowledge, this is the first study demonstrating such relationships between PDEi and glaucoma in a nationally representative sample of the U.S. population.

Interestingly, use of these medications was not associated with lower odds of glaucoma, as we had originally hypothesized. This may be because most of the literature reporting increases in ocular blood flow after administration of sildenafil operated on acute time scales in the range of hours [13–15], whereas glaucoma is a chronic disease and therefore unlikely to be affected by rapid changes in ocular hemodynamics. By studying ≥1 year of sildenafil intake, we attempted to model more long-term associations with glaucoma, and indeed found the opposite effect. Perhaps with repeated exposure to sildenafil, blood is ‘shunted’ away from the ocular circulation in favor of the systemic vasculature, resulting in compromised ocular blood flow and subsequent oxidative injury to the optic nerve head that may be a mechanism for the development or progression of glaucoma. Recent studies showing that acute sildenafil administration lowers blood pressure in a mouse model and in patients with resistant hypertension seem to support this possibility [24,25], although further work is necessary to clarify long-term effects.

The association between theophylline and glaucoma mirrored that of sildenafil in our analysis. While there has been little work regarding the potential effects of theophylline on glaucoma, studies have shown a link between adenosine and IOP. There is evidence that adenosine receptor activation, especially adenosine A1 receptors, lowers intraocular pressure by stimulating the activity of matrix metalloproteinases (MMPs) in the trabecular meshwork, thereby increasing aqueous outflow. This has been demonstrated in animal models [26–28], human trabecular cell lines [29,30], and Phase 2 trials with a novel adenosine A1 receptor agonist [31]. Theophylline’s role in glaucoma may therefore stem from its action as a nonselective adenosine receptor antagonist. Not only could it block aqueous outflow by inhibiting the activation of MMPs, but it has also been implicated in enhancing the reductions in retinal blood flow induced by elevated IOP [32].

In addition to sildenafil and theophylline, other PDEi have been implicated as predictors of higher risk of glaucoma as well. Several studies have shown that heavy caffeinated coffee intake is associated with a higher risk of developing pseudoexfoliation glaucoma or suspected pseudoexfoliation glaucoma compared to those who do not drink coffee [33]. It appears that compounds found in coffee, among them caffeine, elevates plasma homocysteine concentrations after consumption, and hyperhomocystinemia has been associated with pseudoexfoliation glaucoma, perhaps by contributing to the buildup of exfoliation material in the eye [34–37]. Caffeine may further play a role in pseudoexfoliation glaucoma secondary to its non-selective adenosine receptor antagonist and PDEi actions, potentially exacerbating elevated IOP and disrupting normal ocular vasculature via the processes aforementioned.

A major advantage of this study is the use of NHANES, a population-based survey with results generalizable to the civilian, non-institutionalized US. We were also able to account for various potential confounders of the association between PDEi use and glaucoma. However, analyzing NHANES also poses a number of limitations, one of which is the cross-sectional nature of the data. Indeed, we are unable to determine the temporal relationship between PDEi use and glaucoma. Purely observational results are therefore presented, as true causation cannot be determined without longitudinal assessments. We also cannot account for the possible effect of recall bias, as the variables used in our analysis were all self-reported via the NHANES questionnaire.

In addition, glaucoma status was defined by self-report rather than objective measurements from funduscopic exams and photos. Given how the question was phrased, there is a risk self-reported glaucoma includes respondents with ocular hypertension as well. We acknowledge that a diagnosis of glaucoma would ideally be supported by appropriate examination findings and testing. However, though NHANES does provide data on structural and functional parameters of glaucoma, including vertical CDR and visual field loss via FDT testing, the number of respondents with missing data is large, precluding use of these measures to substantiate self-reported cases without severely restricting the study sample size. As well, CDR and FDT testing are themselves imperfect tools in diagnosing glaucoma. While flawed, self-reporting of glaucoma has been shown to be over 96% specific and may underestimate prevalence of disease, suggesting that those who do report a diagnosis of glaucoma likely have the condition [38]. Moreover, there is good to substantial concordance between patient’s self-reported history of glaucoma and a medical record diagnosis [39,40].

Finally, a primary limitation stems from the small number of participants who reported taking sildenafil or theophylline despite the large number of respondents included in the analysis. Limited numbers barred us from studying the relationship of other PDEi with glaucoma as well. Nevertheless, the methodology used to construct NHANES enables us to weight the data to be nationally representative, and our statistically significant regression results identify possible associations that can be the starting point for further investigation.

In summary, this early, exploratory study supports a possible association between sildenafil or theophylline use and glaucoma. We offer two distinct, possible hypotheses for how each medication can be associated with glaucoma–sildenafil through inhibition of PDE5, increase in cGMP, vasodilation, and subsequent shunting of blood away from the ocular circulation while theophylline through nonselective inhibition of adenosine receptors leading to reduced MMP activity and decreased aqueous outflow. If substantiated by further study, prospective randomized trials will need to be performed to determine whether there is a true causal association between PDEi use and glaucoma development and whether use of sildenafil and theophylline can influence the course of disease progression.
